# A case of oral cenesthopathy in which dementia with Lewy bodies developed during treatment

**DOI:** 10.1111/psyg.12541

**Published:** 2020-03-15

**Authors:** Yojiro Umezaki, Takashi Asada, Toru Naito, Akira Toyofuku

**Affiliations:** ^1^ Section of Geriatric Dentistry, Department of General Dentistry Fukuoka Dental College Fukuoka Japan; ^2^ Center for Brain Integration Research Tokyo Medical and Dental University Tokyo Japan; ^3^ Psychosomatic Dentistry, Graduate School of Medical and Dental Sciences Tokyo Medical and Dental University Tokyo Japan

Cenesthopathy is a rare condition characterized by complaints about strange bodily sensations without a corresponding organic cause.^1^ Many patients experience cenesthopathy in the oral region, which is called oral cenesthopathy.^2^ The symptoms vary, with some patients having obviously delusional complaints (e.g. feeling of coils) and others having more mundane complaints (e.g. feeling of excessive mucus secretion, oral stickiness). Some papers have classified cenesthopathy as primary or secondary, and secondary oral cenesthopathy is believed to be a partial symptom of a psychiatric disorder.^3^ Until now, mood disorder and schizophrenia were said to be the main disorders behind secondary oral cenesthopathy, and only a few reports have examined the relationship between oral cenesthopathy and dementia. Herein, we report a case of oral cenesthopathy in which dementia with Lewy bodies (DLB) developed during treatment. Written informed consent was obtained from the patient for publication of this case report and accompanying images.

A 69‐year‐old man, who works as a manager, complained that tape was stuck to his palate for 6 years after the placement of a dental implant. The patient had been examined by his family physician and an otolaryngologist, but no abnormalities corresponding to his complaints were found. He voluntarily visited a psychiatrist but did not get any psychiatric diagnosis and treatment. His family physician then referred him to our clinic.

At the patient's first visit to our clinic, he was well socialized. However, sleep disturbance and depressive mood were observed, but suicidal ideation was not. His Self‐Rating Depression Scale score was 45. On evaluation with the Oral Dysesthesia Rating Scale,^4^ the Symptom Severity Scale score was 12, and the Functional Impairment Scale score was 5, indicating loss of appetite. Considering his clinical history and intra‐oral findings, we diagnosed the patient with oral cenesthopathy. Initially, aripiprazole 0.5 mg/day was prescribed, and the dosage was gradually increased. His symptoms fluctuated but did not improve significantly. Eight months after the first visit, he began to occasionally complain about his forgetfulness and confused our appointment times. Hasegawa's Dementia Scale‐Revised score was administered, and the score was 14; responses were incorrect in the domains of serial 7 subtraction, digits backward, three‐word recall, and category fluency test. Although no significant brain atrophy was detected on magnetic resonance imaging, easy Z‐score imaging system analysis detected decreased cerebral blood flow around the posterior cingulate cortex and precuneus on brain single‐photon emission computed tomography using technetium‐99m ethyl cysteinate dimer **(**Fig. 1 **)**.^5^ After discussions with the patient and his family, we referred him to a neurologist who specializes in dementia. He was diagnosed with DLB according to the criteria suggested by McKeith *et al.,* 6 and rivastigmine was prescribed. Neither cognitive function nor the oral symptom has been further aggravated thus far.

**Figure 1:**
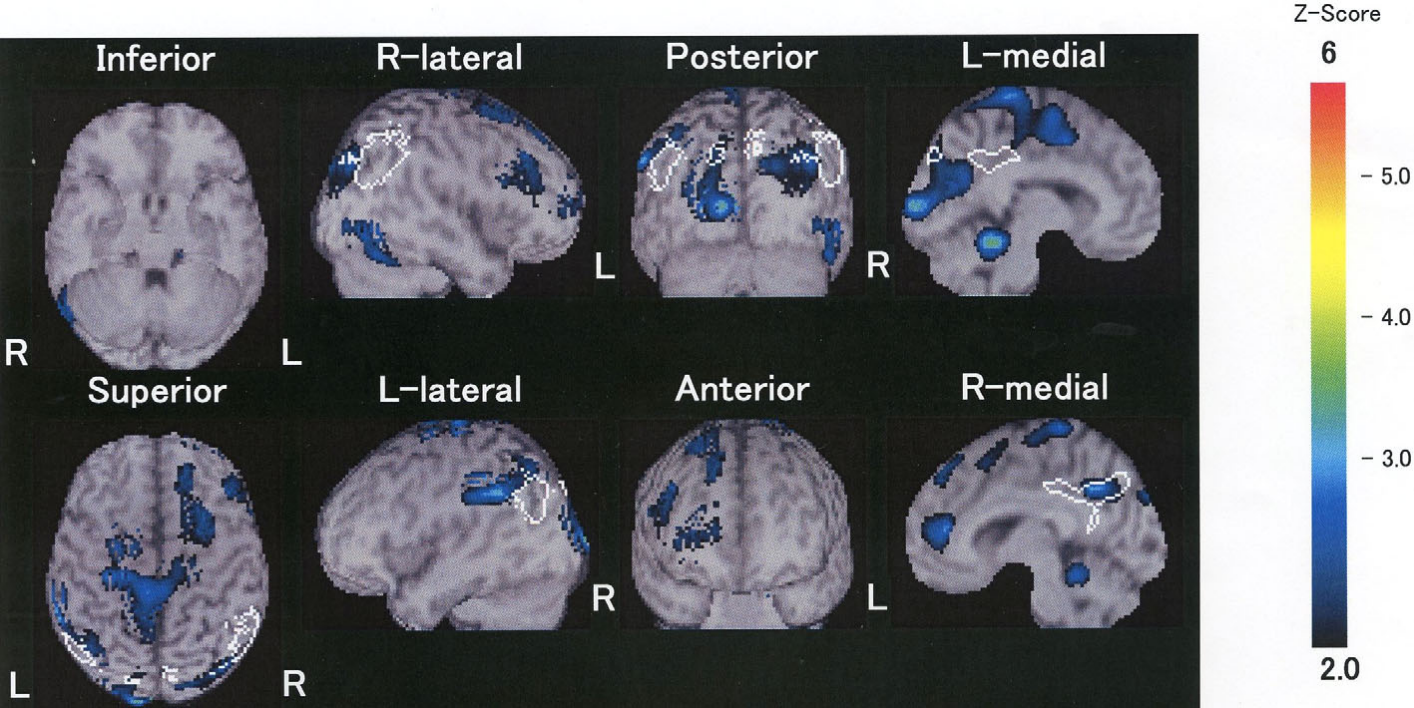
Easy Z‐score imaging system analysis showed hypoperfusion in the posterior cingulate cortex and precuneus. Blue, green, yellow, and red indicate a Z‐score greater than 2.

The core clinical features of DLB are fluctuating symptoms, visual hallucinations, parkinsonism, and sleep behaviour disorders.^6^ Although some reports have found that patients experience auditory and tactile hallucinations as a part of DLB, the present report is the first to report oral cenesthopathy as a secondary symptom of DLB.

Most cases of secondary oral cenesthopathy develop at an early age, and the background disorder is mainly depression, bipolar disorder, or schizophrenia. Only a few studies have reported the relationship between oral cenesthopathy and dementia, including one report concerning the relationship between oral cenesthopathy and Alzheimer's disease.^7^ Thus, in our case, DLB was not suspected at the first visit because the patient's daily life remained the same and elderly onset is uncommon. However, given DLB's features, which can include hallucination, oral cenesthopathy could be a prodromal symptom of DLB. Therefore, a new cognitive function test may be needed to detect hidden dementia, especially in elderly patients with oral cenesthopathy.

The causal relationship between oral cenesthopathy and DLB is still unclear. One possible explanation is that oral cenesthopathy is a secondary symptom of DLB because DLB developed after oral cenesthopathy. Alternatively, the development of DLB could be contingent on oral cenesthopathy. Further investigation is needed to verify these possibilities.

One of the characteristics of DLB is neuroleptic hypersensitivity. Neuroleptic drugs could aggravate parkinsonism and dysautonomia in patients with DLB. Although the risk of such adverse events related to aripiprazole seems to be low because it is a dopamine partial agonist rather than a full antagonist, some studies have reported that aripiprazole could affect the progression of DLB. Therefore, side‐effects should be monitored with the Drug‐Induced Extrapyramidal Symptoms Scale during the treatment of oral cenesthopathy in cases with DLB.

When cognitive dysfunction is not obvious, prodromal dementia patients with oral cenesthopathy tend to persist about receiving dental treatment for oral symptoms. In such cases, treatment adherence could be difficult given that neurologists and dentists do not often cooperate by virtue of their different fields. To prevent patients from discontinuing treatment, oral symptoms should be examined in consideration of cognitive impairment, and cooperation between dentist and neurologist should be initiated as required.

## DISCLOSURE

The authors have no conflicts of interest to declare.

## AUTHOR CONTRIBUTIONS

Y. U. and T. A. drafted the manuscript and prepared the figure. T. N. and A. T. acquired and analyzed the data.
